# Higher- and lower-level processing in strategic reading: Reconceptualising the Survey of Reading Strategies (SORS)

**DOI:** 10.1007/s10339-026-01372-2

**Published:** 2026-07-01

**Authors:** Marcus Warnby

**Affiliations:** Department of Education and Special Education, University of Gothenburg, Gothenburg, Sweden

**Keywords:** Metacognition, Reading strategies, Academic reading, Higher- and lower-level processing, L2 learners, Psychometric validation, Confirmatory factor analysis

## Abstract

The Survey of Reading Strategies (SORS) is widely used to assess English-as-a-second-language (ESL) students’ metacognitive regulation of reading across three strategy dimensions (Global, Problem-Solving, Support), yet this three-factor structure has rarely undergone confirmatory validation. This study introduces *HiLoLev*, a theory-driven two-factor measurement reconceptualisation that distinguishes higher-level integrative/monitoring strategies from lower-level decoding/support strategies. Using confirmatory factor analysis (CFA) on data from 554 Swedish upper-secondary ESL students, legacy three-factor specifications (oblique, higher-order) were contrasted with the proposed theory-driven HiLoLev model based on SORS items. The original structures showed poor fit with high interfactor correlations and substantial cross-loadings; by contrast, an 11-item HiLoLev model fit well (RMSEA = 0.051, SRMR = 0.045) and offered clearer interpretability. While the full SORS remains pedagogically useful, its subscales should not be assumed to represent stable latent constructs for inferential analyses. For research applications (e.g., regression models, mediation analyses, or group comparisons), confirm the structure in-sample via CFA or consider using the 11-item HiLoLev model as a compact, cognitively grounded latent specification of SORS items.

## Introduction

English now permeates instruction and assessment across secondary and higher education. Not only in English-medium programs but also in courses taught in national languages, assigned reading is frequently in English, which is a consequence of the globalisation of academic publishing and Englishisation policies (Macaro et al. 2018; Malmström and Pecorari 2022; Akıncıoğlu 2024). As a result, students in pre-university and university settings must comprehend complex disciplinary texts with limited language scaffolding, heightening cognitive load. This makes strategic regulation of reading, such as planning, monitoring, and compensatory support, especially consequential for English-as-a-second language (ESL) learners across educational phases.

Second-language (L2) reading depends on multiple components, including language competence (Jeon and Yamashita 2024; Laufer and Aviad-Levitzky 2017; Qian 2002) and content knowledge (Koda 2005). A further, widely cited predictor is the use of metacognitive reading strategies, commonly organised into Global, Problem-Solving, and Support (GLOB, PROB, and SUP) dimensions and assessed with the Survey of Reading Strategies (SORS; Mokhtari and Sheorey 2002). Despite its ubiquity (e.g., Aydin and Yildirim 2017; Busby 2018; Caner et al. 2021), the SORS is often used as if these subscales were validated latent constructs across contexts. Confirmatory evaluations are rare, and the dimensional assumptions have not been scrutinised with contemporary psychometric practice; even the developers later called for revision and revalidation (Mokhtari et al. 2018). Although a few studies have raised concerns (Xu et al. 2024; Zhang and Zheng 2020), most research adopts the original three-factor model without testing its assumed structure.

Treating SORS subscale (GLOB/PROB/SUP) mean scores as construct-valid by default risks misleading inferences because of plausible overlap and cross-loadings within and between the intended dimensions. Rather than merely offering a purely technical confirmatory factor analysis (CFA) of the SORS, this study pursues a reconceptualisation of reading strategy dimensions grounded in cognitive reading theory, distinguishing higher- from lower-level processing. Higher-level strategies involve integration, monitoring, and evaluation; lower-level strategies scaffold decoding and local processing. This reconceptualisation is implemented through a theory-driven reframing of the latent structure underlying SORS items, rather than just a revision of the instrument itself.

Using data from 554 Swedish upper-secondary ESL students preparing for university studies, the internal structure of the 30-item SORS is examined via confirmatory factor analysis (CFA). Two widely assumed legacy specifications are evaluated (an oblique three-factor model and a higher-order variant), and contrasted with a theory-driven two-factor alternative (HiLoLev) derived from SORS items. This model separates higher-level integrative and monitoring strategies from lower-level decoding and support strategies, aligning the measurement with a long-standing distinction in cognitive accounts of comprehension.

The goal is a parsimonious, theoretically grounded, and psychometrically defensible structure that can underpin substantive research on links between strategy use and learner characteristics (e.g., proficiency, age, gender). Beyond instrument housekeeping, clarifying these two functions matters for cognitive science because it separates variance related to building and monitoring a coherent representation of text from variance related to alleviating local processing demands, which are processes that are conceptually distinct and may diverge in their relations to outcomes.

This study therefore (i) evaluates whether the original three-factor structure holds under CFA, (ii) introduces and tests a two-factor model aligned with cognitive reading theory on the full item pool, and (iii) proposes a transparent, theory-driven 11-item short form (HiLoLev) for latent measurement. Importantly, the present study proposes a measurement model for research use, rather than a full revision of the SORS.

## Theoretical framework

Reading comprehension in a second language (L2) is a cognitively demanding activity shaped by multiple interacting components. These include linguistic competence (e.g., vocabulary and grammar), cognitive processes operating at various levels (e.g., decoding, inference-making), and the deployment of reading strategies that enable readers to monitor and regulate their understanding (Grabe and Stoller 2019; Jeon and Yamashita 2024).

Among these, vocabulary knowledge is consistently found to be the most central linguistic predictor of L2 reading comprehension (e.g., Qian 2002; Laufer and Aviad-Levitzky 2017). Grammatical knowledge also has a moderate unique effect (Jeon and Yamashita 2024), but as Laufer and Aviad-Levitzky (2017, p. 730, footnote 1) observe, there is a lack of evidence that “L2 learners with poor (below threshold) vocabulary achieved good reading scores because of good grammatical knowledge.”

In addition to language competence, reading comprehension depends on readers’ motivation, content knowledge, and the use of metacognitive reading strategies. Unlike automatic decoding processes, reading strategies are intentional actions aimed at facilitating understanding, particularly in the face of textual difficulty or ambiguity (Afflerbach et al. 2008). In the context of L2 reading, certain strategies may serve a compensatory role when linguistic knowledge is insufficient, or optimise comprehension through planning, monitoring, and evaluation. In contrast to automatic processes, strategies involve deliberate and effortful decisions to overcome problems or enhance understanding (Afflerbach et al. 2008; Pressley and Afflerbach 1995).

To assess learners’ awareness and reported use of such strategies, some instruments aim to capture academic reading as a multifaceted competence, including, for example, reading strategies, genre awareness, and content knowledge (e.g., Nejadghanbar et al. 2024). Others focus more narrowly on metacognitive reading strategies in academic contexts. Given that English continues to function as the global lingua franca of academia, where ESL students frequently and increasingly encounter English-language course materials even when instruction is conducted in the local majority language, a targeted instrument for this learner group becomes essential. A recent example of such an instrument development is the English for Educational Reading Strategies Inventory (Wilawan 2022) which draws on cognitive theory of academic reading. However, the most widely used tools in this context is the Survey of Reading Strategies (SORS; Mokhtari and Sheorey 2002), developed specifically to capture ESL students’ strategic engagement with academic and school-related texts across three dimensions: (i) global, (ii) problem-solving, and (iii) support strategies.

### The survey of reading strategies (SORS)

With respect to the argument that reading demands strategic engagement with the text, Mokhtari and Sheorey (2002) developed the Survey of Reading Strategies (SORS) as a self-report instrument for assessing ESL students’ metacognitive awareness and use of reading strategies when engaging with academic or school-related materials in English. The instrument was adapted from the Metacognitive Awareness of Reading Strategies Inventory (MARSI; Mokhtari and Reichard 2002), originally designed for first language (L1)^1^ readers.

The SORS includes 30 items rated on a 5-point Likert scale and is structured around three dimensions:

1. Global reading strategies (GLOB): 13 items that are assumed to reflect students’ self-regulatory strategies to “monitor and manage their reading” (p. 4), referring to intentional strategies related to planning text comprehension, such as setting reading goals, previewing text, and assessing understanding.
2. Problem-solving strategies (PROB): 8 items assumed to capture adaptive and responsive strategies applied when the text “becomes difficult or easy” (p. 4), including localised and focused strategies such as adjusting the reading speed, visualising, and regulating attention.
3. Support strategies (SUP): 9 items assumed to represent basic and externally oriented strategies, such as using a dictionary, paraphrasing, or reading aloud, which support comprehension without involving deeper, integrative processing of the text.

While the SORS is widely used, its development history is opaque, marked by inconsistent terminology, shifting item structures, and limited psychometric validation. A key confusion concerns reliance on MARSI statistics (Mokhtari and Reichard 2002): MARSI was developed and validated through exploratory factor analysis with L1 English students (*N* = 825), and yielded GLOB (α = 0.92), PROB (α = 0.79), and SUP (α = 0.87) with overall α = 0.93. These coefficients are often cited in SORS studies despite coming from a different instrument and population (e.g., Busby 2018; Malcolm 2009). In a later revision (MARSI-R), Mokhtari et al. (2018) confirmed a three-factor structure via CFA for L1 readers but explicitly noted that the SORS still required revision and revalidation with its L2 target population: “we intend to revise and revalidate [the SORS] to follow the practice of the MARSI as well” (p. 239). Thus, although the three-factor structure has received confirmatory support for MARSI-R in L1 contexts, SORS has not undergone comparable CFA-based validation with L2 readers. Simply referring to psychometric properties established for an L1 instrument does not justify assuming equivalent validity for an L2 instrument, particularly given that lower-level processing demands are more salient in L2 reading and may alter the functional organisation of reported strategies.

In contrast to the MARSI, the first SORS (Sheorey and Mokhtari 2001) was intended for both L1 and L2 readers from high school through college, with 28 items under Metacognitive (10), Cognitive (12), and Support (6) labels and a total reliability of α = 0.89 in an ESL sample; however, subscale reliabilities and factor analyses were not reported.

By 2002, the authors introduced a 30-item SORS targeted at ESL learners, now categorised into GLOB, PROB, and SUP to mirror MARSI’s framework. No new reliabilities or factor analyses were reported for this revision; instead, MARSI values were referenced, implicitly assuming that L1 psychometrics applied to an L2 instrument and that item changes would not affect internal consistency. Notwithstanding these issues, Mokhtari and Sheorey (2002) outlined theoretical rationales and practical uses.

Rather than collapsing strategy use into a single score, Mokhtari and Sheorey (2002) proposed a three-factor model, describing GLOB/PROB/SUP as “three broad categories of reading strategies” (p. 4), and referring to previous MARSI statistics claimed them to be theoretically and psychometrically reliable (p. 3–4). At the same time, they consistently referred to these as “subscales” (p. 4) and emphasised that skilled readers flexibly coordinate strategies across categories, stating: “they know which strategies to use and how to use them to ensure access in reading comprehension” (p. 7). This duality leaves open whether the SORS represents three correlated constructs or facets of a general factor.

**Fig. 1:**
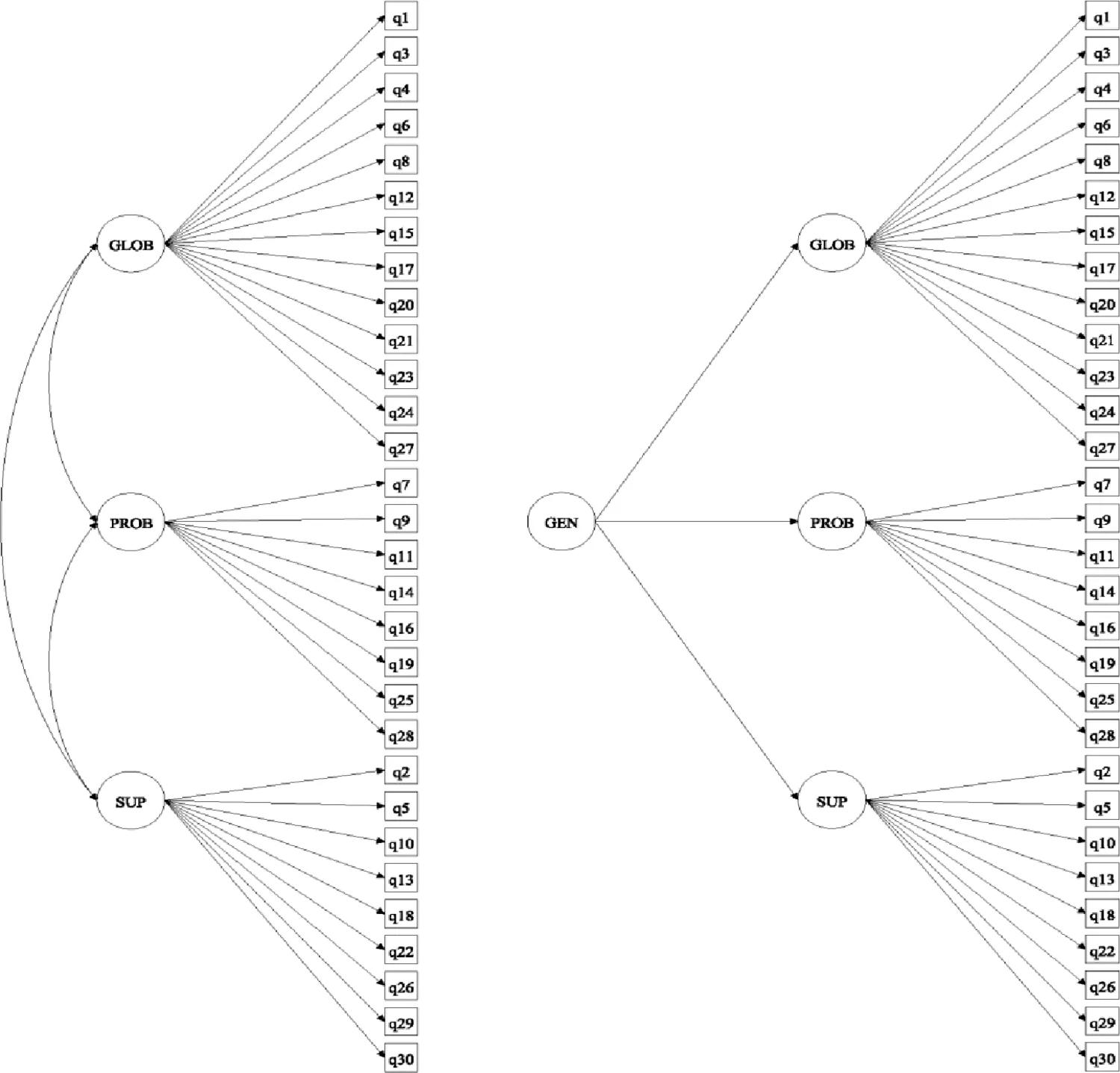
Two conceptual factor models of the SORS: (i) oblique, (ii) higher-order

Accordingly, two plausible structural interpretations of the SORS follow based on the original descriptions by Mokhtari and Sheorey (2002): an oblique three-factor model with correlated but distinct latent variables, and a higher-order model with a general metacognitive factor underlying GLOB/PROB/SUP. Both are tested here (Fig. 1).

Mokhtari and Sheorey (2002) recommend that the SORS be administered alongside background questions “requesting information about [students’] age, gender, and self-rated ability in reading English” (p. 4), and suggest complementing the instrument with proficiency estimates “obtained on a standardized test like the Test of English as a Foreign Language (TOEFL)” (p. 4). They advise computing subscale means for GLOB, PROB, and SUP by averaging item responses on the 5-point Likert scale (1 = “never/almost never” to 5 = “always/almost always”), which are then used for group comparisons or correlations. However, this practice presumes unidimensional subscales. Without empirical verification of the internal structure, aggregating items may obscure cross-pressure or cancel divergent item behavior, especially given item revisions and the shift from L1 to L2 contexts. Consequently, SORS subscale means may not reliably index distinct constructs.

Although the SORS was originally developed for ESL learners reading in English, it has since been translated and used in multiple linguistic contexts, including Arabic, Turkish, and Indonesian (e.g., Aydin and Yildirim 2017; Malcolm 2009; Mardiningrum and Salsabilla 2022). However, such adaptations have rarely been accompanied by systematic dimensional structural validation, and translated versions or cross-linguistic applicability were never addressed in the original work (Mokhtari and Sheorey 2002).

In summary, the SORS’s pedagogical premise may be sound, but its structural assumptions remain under-examined. Even the developers acknowledge the need for SORS revision and revalidation (Mokhtari et al. 2018). The present study responds by testing theoretically motivated CFA models (Fig. 1) to evaluate whether the original tripartite structure holds, whether a general factor is implicated, or whether an alternative configuration better reflects L2 reading strategy dimensions. The next sections review empirical uses of the SORS and introduce an alternative, cognitively grounded two-factor model.

### Previous uses and limitations of the SORS

Despite its widespread use, the SORS is rarely subjected to factor-analytic validation prior to substantive analysis. Most studies simply adopt the original three-factor structure (GLOB, PROB, SUP) and compute subscale means to explore group differences or correlations. For instance, gender-based comparisons have been reported in Turkey (Caner et al. 2021) and Indonesia (Mardiningrum and Salsabilla 2022), while Aydin and Yildirim (2017) examined relative subscale use among Turkish undergraduates. Other studies relate strategy use to vocabulary knowledge (Wang et al. 2022), academic discipline (Caner et al. 2021), or L1–L2 context differences (Busby 2018). Proficiency group comparisons are particularly common, with findings indicating that higher-proficiency students report more frequent use of global and problem-solving strategies, whereas lower-proficiency students tend to rely more on support strategies (e.g., Malcolm 2009; Zhang and Wu 2009; Busby 2018). These trends are consistent with the view that skilled readers emphasise planning/monitoring, while less skilled readers depend more on local, externally supported strategies. Across both full (30-item) and shortened/adapted versions, however, a shared limitation remains: few studies conduct factor analysis to verify whether the assumed subscales are coherent and distinct. Instead, raw means are treated as proxies for latent constructs without first establishing structural validity within the sample. Consequently, this widespread reliance on untested subscales raises doubts about the reliability of such claims, not because the findings are necessarily incorrect, but because they rest on unvalidated measurement assumptions.

Only a handful of studies have challenged the assumed three-factor structure of the SORS. Zhang and Zheng (2020) tested a translated SORS with Chinese senior high school students using EFA and CFA and found that the hypothesised GLOB/PROB/SUP structure was not supported. Instead, they reported very high interfactor correlations and concluded, after substantial item reduction, that a unidimensional solution provided a better statistical fit, resulting in a 16-item general-factor version. While this data-driven revision is informative in demonstrating the lack of discriminant validity among the three original subscales, it leaves open how different reading strategies may function at different levels of processing. Notably, Zhang and Zheng explicitly call for further validation work to clarify the construct dimensionality of the SORS across contexts. Their findings align with the present study in questioning the stability of the original three-factor model and motivate a theoretically grounded reconceptualisation.

Another, more radical revision is seen in Xu et al. (2024), who, with Chinese university EFL learners, also removed several items, added new items and regrouped them into two theoretically motivated categories (globally oriented strategies versus more local, support-oriented strategies). This suggests potential benefits of structural revision, but their revised categorisation was not validated via factor analysis. Despite different aims, both Zhang and Zheng (2020) and Xu et al. (2024) raise doubts about whether the original SORS subscales operate as distinct dimensions.

Together, these studies indicate that the SORS structure is not universally stable across contexts. They do not undermine the pedagogical utility of the prompts, but they do raise legitimate concerns about treating GLOB/PROB/SUP means as distinct constructs without prior validation. These concerns echo Mokhtari et al.’s (2018, p. 239) own recommendation to “revise and revalidate” the SORS framework.

### Strategies for higher-level and lower-level reading processes

**Fig. 2:**
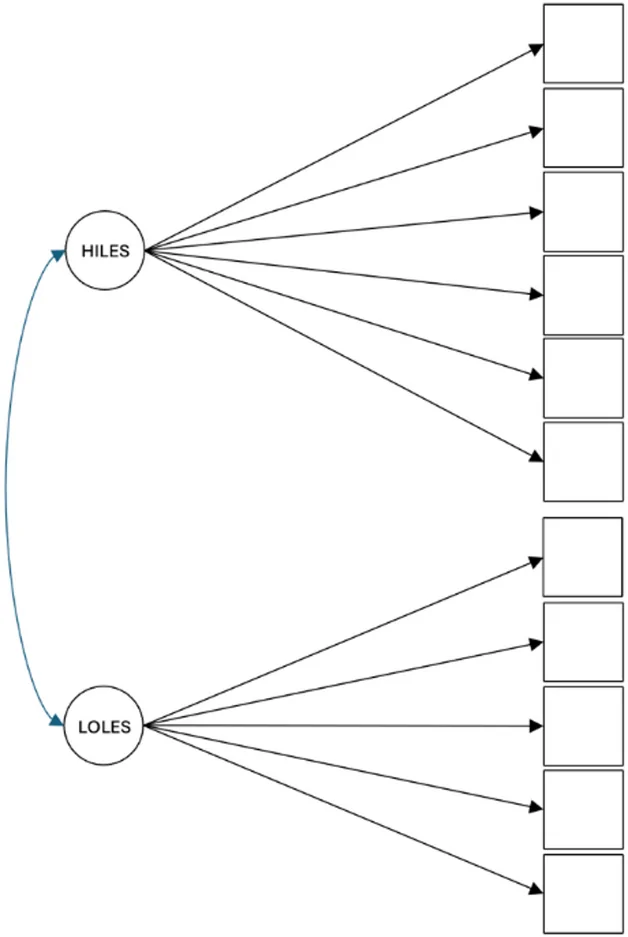
A two-factor HiLoLev model illustration, distinguishing between higher-level (HILES) and lower-level (LOLES) reading strategies

In cognitive models of reading, a well-established distinction is between lower-level and higher-level processes (Grabe and Stoller 2019). Lower-level processes are automatic, foundational operations (decoding, word recognition, syntactic parsing) that support access to literal meaning with minimal effort. Higher-level processes involve constructing and updating a situation model (integration, inference, evaluation, critical reflection) and typically recruit metacognitive regulation, especially under ambiguity or difficulty. When lower-level fluency is weak, working-memory demands rise and constrain higher-level integration, highlighting reading as a multi-level cognitive activity in which foundational fluency supports advanced meaning-making. This section develops a distinction between higher- and lower-level reading strategies, summarised schematically in Fig. 2 and later formalised as the *HiLoLev* model.

This two-level framework maps neatly onto strategic behaviour in reading. In the present study, the term reading processes refers to the cognitive operations involved in comprehension itself (e.g., decoding, integration, inference-making), whereas reading strategies refer to deliberate, self-regulatory behaviours that readers use to support, monitor, or compensate for those processes.

Strategies that support lower-level processing, such as rereading, dictionary use, paraphrasing (often L1 rewording), translating, help readers overcome lexical or syntactic difficulty and are used when surface comprehension is under strain. These lower-level strategies (LOLES) function as compensatory scaffolds when automaticity is insufficient. Their distinctiveness is consistent with evidence that lexical resources (e.g., vocabulary size, collocational competence) make additive contributions to L2 performance, helping explain why local support strategies increase when linguistic resources are limited (Masrai 2023). LOLES broadly align with SORS Support strategies and with word-/text-level decoding aids in Xu et al. (2024).

By contrast, higher-level strategies (HILES) are goal-directed cognitive–metacognitive behaviors that maintain coherence and build a global representation of the text, e.g., setting goals, previewing/predicting, generating inferences, evaluating arguments, synthesising across sections. In the SORS, these map most closely to Global and Problem-Solving assumptions. Such actions are hallmarks of constructively responsive and skilled reading (Pressley and Afflerbach 1995; Afflerbach et al. 2008). Recent L2 evidence using verb-clustering (Tang et al. 2025) shows that readers monitor protagonists, time, and space, and that individual differences in visual imagery facilitate coherence maintenance, consistent with HILES as integration/monitoring processes.

This functional split mirrors Mokhtari and Sheorey’s (2002, p. 4) observation that skilled readers flexibly use global/problem-solving behaviors, whereas less proficient readers “tend to expend more time and energy struggling with individual words.” Although they did not propose a two-factor model, the contrast motivates a restructuring in which HILES index global comprehension control and LOLES index compensatory decoding support. Frequent LOLES use may signal limited processing capacity or working-memory overload that hinders deeper comprehension, particularly in L2 reading. Accordingly, SORS data may be modeled more fruitfully with two interpretable factors than as a single dimension (cf. Zhang and Zheng 2020) or as three inherently distinct subscales without empirical verification (cf. Mokhtari and Sheorey 2002). This view also accords with indications that higher-proficiency readers report greater use of global/problem-solving strategies, while lower-proficiency readers rely more on support strategies (e.g., Busby 2018; Zhang and Wu 2009; Malcolm 2009). Recent accounts characterise high-level comprehenders as flexibly deploying strategies across levels; this functional flexibility likely contributes to an expected positive intercorrelation between HILES and LOLES, as skilled readers mobilise both sets situationally (Wu et al. 2023). Reported reading strategies are treated not as direct measures of cognitive processes, but as theoretically motivated proxies for how readers regulate processing at different levels during reading (Pressley and Afflerbach 1995; Grabe and Stoller 2019).

The idea of grounding strategy frameworks in cognitive theory is not unique to the present study. For example, Wilawan (2022) developed a 27-item instrument aligned with academic reading processes, replacing SORS’s broad subscales with ten granular dimensions (goal setting, text structure awareness, critical evaluation, etc.). While theoretically rich, its breadth may limit parsimony and generalisability. In contrast, a higher–lower distinction preserves cognitive coherence while remaining concise and interpretable within multilevel comprehension models.

In sum, theoretical and empirical considerations suggest that SORS may benefit from conceptual and structural revision. The present study therefore proposes a theory-driven two-factor specification, henceforth referred to as the *HiLoLev* model (Fig. 2), which is evaluated via CFA alongside previously posited models to assess whether it yields a more interpretable and psychometrically robust structure.

The HiLoLev model is intended to serve both conceptual and practical purposes. Conceptually, it represents a theory-driven reconceptualisation of reading strategies by relating them to higher- and lower-level cognitive processes involved in reading comprehension. The distinction does not imply that strategies themselves constitute those reading processes; rather, strategies are understood as behaviours that support cognitive processing at different levels of the reading process. At the measurement level, HiLoLev provides a principled way of reorganising existing SORS items as indicators of these two functional dimensions, enabling their use in latent variable modelling. Practically, the resulting 11-item specification offers a compact option for re-analysing existing SORS datasets or for use in future research where a parsimonious, theoretically grounded strategy model is required.

### Purpose and research questions

Given the widespread use of SORS in ESL research, it is essential to determine whether its three-factor structure is valid across different learner populations. The original developers, Mokhtari and Sheorey (2002), did not conduct any confirmatory factor analysis (CFA) or compare alternative measurement models, leaving the three-factor structure empirically unverified. The present study addresses this gap by evaluating the structural validity of SORS via CFA with a sample of Swedish university-preparatory ESL students.

Specifically, this study aims to answer the following research questions:

1. Does the three-factor structure of SORS with its claimed distinct factors GLOB, PROB, and SUP hold in CFA, indicating that it is a stable measurement instrument, and how does this three-factor oblique model behave in comparison to a higher-order model including a common general reading strategy factor?
2. What is the empirical and theoretical validity of a reduced two-factor model of the SORS, derived from reading process theory and distinguishing between higher-level and lower-level strategies?

## Method

### Participants

The study included 554 Swedish upper secondary school students in their final year of university-preparatory education. All had passed the final mandatory English course required for university eligibility in Sweden (designed to ensure CEFR B2). Participants were 17–20 years old (*M* = 18.05, *SD* = 0.22) and all were L2 English learners.

Participation was voluntary with informed consent. Data were collected in classrooms during regular instruction time using a paper-and-pencil format. The study was approved by the Swedish Ethical Review Authority (Dnr 2019–04470).

Swedish upper-secondary curricula explicitly reference reading strategies, for example, the English syllabus specifies that instruction should address “strategies for understanding details and drawing conclusions about content and messages, for example by visualising, associating, retelling, predicting content and asking questions” (Skolverket 2011, *np; author’s translation*). This suggests that students were familiar with both cognitive and metacognitive notions of strategy. Given the prominent role of English in Swedish higher education (Malmström and Pecorari 2022), this sample is relevant for examining ESL students’ strategic preparedness for academic reading.

### Instruments

#### Survey of reading strategies (SORS)

The 30-item SORS (Mokhtari and Sheorey 2002) with its 5-point Likert was translated into Swedish focusing on functional/construct fidelity rather than literal wording. A Swedish L1 teacher reviewed the translation, and it was piloted in three classroom rounds with upper-secondary ESL students. Only minor wording tweaks were required. Item wording is given in Appendix 1, and descriptive statistics for all 30 SORS items are reported in Appendix 2.

### Data analysis

To examine the structural validity of the SORS, a series of confirmatory factor analyses (CFAs) were conducted in Mplus Version 8.11 (Muthén and Muthén 1998). Items were treated as approximately continuous 5-point indicators. Item distributions were acceptable for CFA with MLR: skewness ranged from − 0.82 to 0.87 and excess kurtosis from − 1.23 to 0.31; *N*s varied slightly by item (min = 486, max = 554) (Appendix 2). Models were estimated with robust maximum likelihood (MLR), which provides robust standard errors and a scaled χ².

The following theoretically motivated CFA models were tested and compared:

*Model 1*: Oblique three-factor model. The original SORS structure (Mokhtari and Sheorey 2002) with three correlated latent factors: GLOB, PROB, and SUP.

*Model 2*: Higher-order model. A second-order general reading-strategies factor (GEN) explaining correlations among GLOB/PROB/SUP.

*Model 3*: Two-factor HiLoLev model. A theory-driven reconfiguration distinguishing higher-level strategies (HILES) and lower-level strategies (LOLES), first with all items and then as a short form.

Model fit was evaluated with CFI, RMSEA (90% CI), and SRMR, interpreted jointly (Hu and Bentler 1999; Kline 2016). As guidelines, CFI ≥ 0.95 indicates good (≈ 0.90–0.95 acceptable); RMSEA ≤ 0.06 good (≈ 0.06–0.08 acceptable); SRMR ≤ 0.05 good (≤ 0.08 acceptable). For the retained model, we report standardised loadings and modification indices used only for diagnostics. Missing data were handled with full-information ML under MLR.

### Item-selection pipeline

All 30 SORS items were first mapped a priori to HILES vs. LOLES based on cognitive theory (integration/monitoring vs. decoding/support). The full 30-item two-factor model was fitted to enable fair comparison with the three-factor baseline. We then applied three pre-specified pruning rules: (i) retain items with λ ≥ 0.45 and *R*² ≥ 0.20 on the theorised factor; (ii) drop items showing substantive cross-pressure (StdYX EPC ≥ 0.30) or local dependence (MI ≥ 30) with close paraphrases; (iii) within overlapping content clusters, keep the theoretically clearest representative. Where item classification was theoretically ambiguous, decisions were informed by theory, empirical diagnostics, and parsimony rather than by mechanical criteria alone. This yielded an 11-item HiLoLev set (6 HILES; 5 LOLES). Importantly, post hoc decisions were limited to item-level retention or exclusion and did not alter the a priori theoretical definition of the HILES and LOLES constructs. Full item-level diagnostics and pruning rationales are provided in Appendix 3. Accordingly, this study operationalises HiLoLev as an 11-item short form for latent-variable work; the 30-item two-factor model is retained mainly as a diagnostic benchmark.

## Results

The results are organised into two sections, each corresponding to one of the research questions. Before addressing each research question in detail, Table 1 presents a comparative summary of model fit indices for the tested models.

**Table 1:** Model fit comparisons

Model	χ² (df)	RMSEA (90% CI)	CFI	TLI	SRMR
Model 1: Oblique 3-factor	1737.299 (402)	0.077 [0.074, 0.081]	0.639	0.609	0.077
Model 2: Higher-order 3-factor	1737.297 (402)	0.077 [0.074, 0.081]	0.639	0.609	0.077
Model 3a: 30-item 2-factor model	1553.193 (404)	0.074 [0.071, 0.078]	0.665	0.639	0.072
Model 3b: 11-item HiLoLev model	130.470 (43)	0.051 [0.039, 0.064]	0.930	0.910	0.045

### Assessing the original three-factor structure

#### Model 1. Oblique model

An oblique three-factor model, consistent with Mokhtari and Sheorey’s (2002) original conceptualisation of Global, Problem-Solving, and Support strategies, was first assessed (see Table 1). All indicators loaded significantly in the expected direction (all *p* < .001), but the overall model fit was marginal to poor: χ²(402) = 1737.299, RMSEA = 0.077 (90% CI [0.074, 0.081]), CFI = 0.639, TLI = 0.609, SRMR = 0.077. Thus, the theorised structure does not reproduce the observed covariances well.

Several features point to structural weaknesses. First, the interfactor correlations were very high (GLOB–PROB: *r* = .835; GLOB–SUP: *r* = .806; SUP–PROB; *r* = .704), indicating substantial multicollinearity and limited discriminant validity among the three constructs. Second, modification indices suggested that many items cross-loaded or shared excess covariance. For example, q23 (a GLOB item) showed a cross-loading tendency on PROB (MI ≈ 20.008); q19 (PROB) showed a strong pull toward SUP (MI ≈ 40.861); and q26 (SUP) showed a strong signal on GLOB (MI ≈ 41.071). In addition, several item pairs showed local dependence (e.g., q30–q29, MI ≈ 33.199; q28–q24, MI ≈ 40.455), consistent with redundancy in content.

Finally, although standardised loadings were statistically significant, several were modest (e.g., q28 λ = 0.259; q29 λ = 0.292; q30 λ = 0.239), suggesting that a sizeable proportion of their variance was not explained by the intended factor. These findings mirror those of Zhang and Zheng (2020), who reported similar fit for the original three-factor model in a Chinese secondary EFL sample (RMSEA = 0.079, CFI = 0.819). Together, these results call into question the theoretical distinctiveness of the three-factor model. Rather than measuring unique dimensions, many items seem to tap into a broader, general reading strategy tendency, motivating the test of a higher-order model.

#### Model 2. Higher-order model

Imposing a single higher-order factor (GEN) produced the same global fit as the oblique model (χ², RMSEA, CFI, TLI, SRMR unchanged). The three first-order factors loaded strongly on GEN (GLOB λ = 0.978; PROB λ = 0.854; SUP λ = 0.824), indicating substantial common variance and supporting the idea of an overall strategy tendency. However, the residual variances for PROB (ψ = 0.271, *p* < .001), and SUP (ψ = 0.321, *p* < .001) remained significant, suggesting that meaningful unique variance exists in these two first-order constructs even after controlling for GEN. In addition, the factor loadings within each first-order factor revealed a generally acceptable pattern with loadings ranging from 0.385 to 0.587 for GLOB items, from 0.259 to 0.571 for PROB, and from 0.239 to 0.615 for SUP. Yet crucially, the MIs again revealed sizeable cross-loadings that questioned the factorial distinctiveness of the three subscales (e.g., q19→SUP, MI ≈ 40.859; residuals q30–q29, q27–q23, q14–q9). In short, the higher-order model captures the shared core but does not resolve problematic cross-loadings or local dependencies.

Taken together, these findings point to the limitations of the original three-factor specification and suggest that an alternative conceptualisation, such as a two-factor model grounded in cognitive reading theory, may offer a more robust representation of the SORS structure if problematic items are carefully handled.

### Proposing a new two-factor model based on reading processes

#### Model 3a: The 2-factor model

A two-factor model with all 30 items was also fitted to allow a fair global fit comparison and to examine where the scale struggles conceptually (given several items are hard to place). As expected, that broad model also showed limited fit (e.g., RMSEA=0.074, CFI=0.665, TLI=0.539, SRMR=0.072) and multiple cross-loading/LD suggestions. Its somewhat better fit than the original 3-factors baseline nevertheless supports a two-factor structure for SORS-type content; however, the full pool contains indicators of similar wordings increasing the MIs and lacks sufficiently clean indicators to use the factors as distinct latent variables in SEM. Hence a final model is presented reducing the numbers of items: the 11-item HiLoLev model, which retains the conceptual split while meeting stricter psychometric criteria for distinct factors. (See Appendix 3 for more details and pruning)

#### Model 3b: The HiLoLev model

Building on the overlap and misfit seen in earlier specifications (and the acceptable-only performance of the 30-item two-factor version; Model 3a), we tested a theory-driven HiLoLev solution that separates higher-level strategies (HILES), such as integration and comprehension monitoring, from lower-level strategies (LOLES), such as decoding and local support. This distinction maps onto widely studied processing levels in reading: discourse-level integration and executive monitoring versus local operations and external supports. In contrast, the original SORS categories (GLOB/PROB/SUP) mix processes across levels (e.g., monitoring and speed adjustment with support behaviors), which likely contributed to the cross-factor signals observed earlier.

Eleven indicators were retained a priori to maximise conceptual coverage while minimising cross-factor interference: six HILES (q3, q9, q11, q14, q20, q21) and five LOLES (q2, q5, q10, q13, q18). Table 2 lists item wording and factor belonging.

**Table 2:** Item composition of the final 11-item HiLoLev model, including item wording

Factor	Item	Item wording
HILES	q3	I think about what I know to help me understand what I read
	q9	I try to get back on track when I lose concentration
	q11	I adjust my reading speed according to what I am reading
	q14	When text becomes difficult, I pay closer attention to what I am reading
	q20	I use typographical features like boldface and italics to identify key information
	q21	I critically analyze and evaluate the information presented in the text
LOLES	q2	I take notes while reading to help me understand what I read
	q5	When text becomes difficult, I read aloud to help me understand what I read
	q10	I underline or circle information in the text to help me remember it
	q13	I use reference materials (e.g., a dictionary) to help me understand what I read
	q18	I paraphrase (restate ideas in my own words) to better understand what I read

**Fig. 3:**
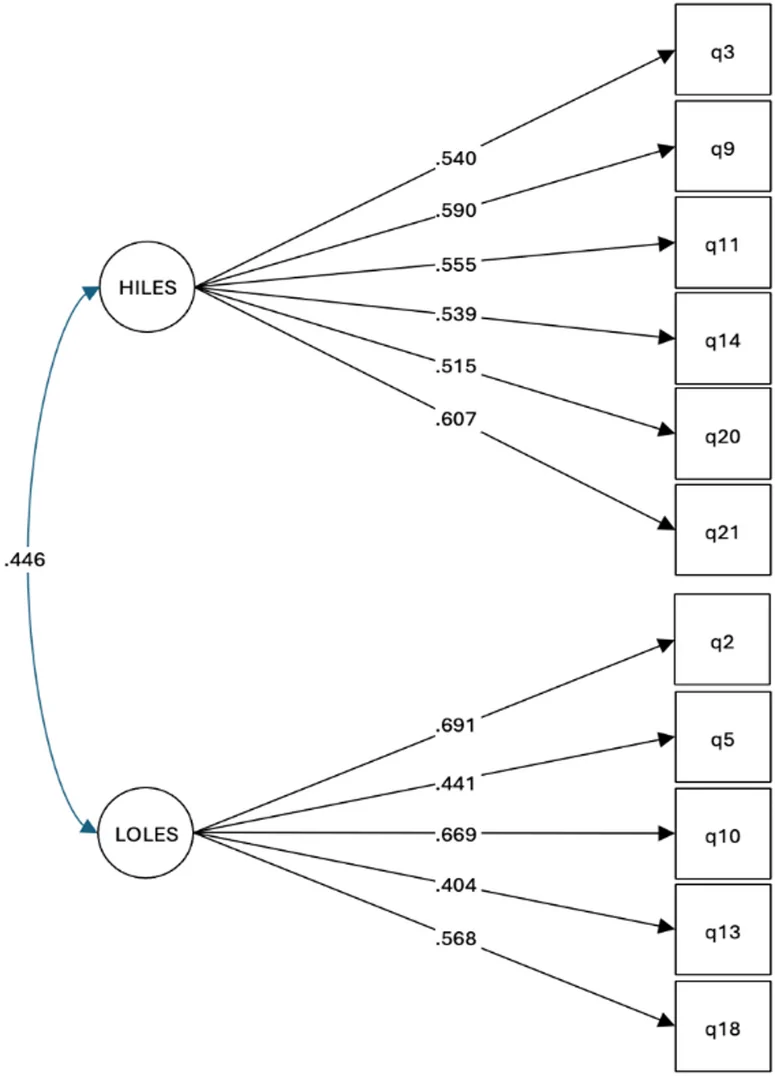
The HiLoLev model with standardised factor loadings

The HiLoLev model fit well, χ²(43) = 106.130, *p* < .001, RMSEA = 0.051 (90% CI [0.039, 0.064]), CFI = 0.930, TLI = 0.910, SRMR = 0.045. All items loaded significantly on their intended factor (*p* < .001); HILES loadings ranged 0.515–0.607, LOLES 0.404–0.691. The latent correlation was *r* = .446 (*p* < .001), indicating related but clearly distinguishable constructs. A few small modification indices suggested content-adjacent residual associations (e.g., q14–q9). To preserve a simple, theory-consistent structure and avoid local dependence, no cross-loadings or correlated residuals were freed. Figure 3 shows standardised loadings. Model-based reliability was satisfactory (ω (HILES) =0.73, ω (LOLES) =0.69), indicating adequateinternal consistency of the indicators within the specified CFA model.

The HiLoLev structure yields a compact, psychometrically clean measure that aligns theoretically with core mechanisms in reading such as executive control/monitoring for meaning construction versus local, support-oriented behaviors. Practically, this reduces construct overlap present in the legacy SORS subscales and provides factors that are more interpretable for subsequent cognitive analyses (e.g., relating higher-level monitoring to comprehension outcomes while distinguishing it from local note-taking or dictionary use).

## Discussion and conclusion

This study set out to evaluate the factorial structure of the widely used SORS, originally proposed as a three-factor instrument comprising GLOB, PROB, and SUP strategy dimensions. Using CFA in a sample of Swedish university-preparatory ESL students, the original tripartite structure showed poor fit and limited discriminant validity; none of the models retaining three strategy dimensions yielded acceptable fit. In contrast, a theoretically grounded two-factor 11-item model, HiLoLev, demonstrated good fit and interpretability. Conceptually, this separates variance attributable to higher-level integration/monitoring (HILES) from variance attributable to local decoding support (LOLES), thereby reducing construct bleed present in GLOB/PROB/SUP and yielding cleaner constructs for cognitive modelling. These findings support a reconceptualisation of the SORS that better aligns with cognitive models of reading comprehension processes.

While the pedagogical value of the SORS is not in question, its continued use as a psychometric instrument demands scrutiny. The original three-dimensional structure has frequently been adopted without prior validation, often by calculating subscale means for GLOB, PROB, and SUP and using these in group comparisons or regression analyses. However, as this study, in line with Zhang and Zheng (2020), has shown, several items exhibit cross-pressure and fail to cluster cleanly into the intended categories. Using unvalidated subscale scores risks misinterpretation, particularly when such scores form the basis of claims about gender or proficiency differences. These results echo the validity concerns raised by Mokhtari et al. (2018) and reinforce the need to reassess the SORS’ dimensional logic. A practical implication for revisiting previously collected SORS data is straightforward: confirm the structure in-sample before inferential use; where feasible, consider the HiLoLev specification as a more parsimonious and theoretically coherent alternative.

The two-factor HiLoLev model addresses these concerns by regrouping a subset of SORS items into higher-level strategies (HILES), which support global integration and self-monitoring, and lower-level strategies (LOLES), which aid surface-level processing. This distinction is grounded in cognitive models of L2 reading (Grabe and Stoller 2019) and supported by empirical trends showing that higher-proficiency readers tend to use global and problem-solving strategies more frequently, while less proficient readers rely more on support strategies such as paraphrasing or dictionary use (e.g., Busby 2018; Zhang and Wu 2009). The moderate positive correlation between HILES and LOLES in the final model (*r* = .446) is theoretically expected given that skilled readers flexibly use strategies across levels as task demands shift, which naturally induces intercorrelation. Although the shared variance across SORS items indicates a general tendency in reported strategy use (cf. Zhang and Zheng 2020), the present study does not adopt a unidimensional representation, as a single factor would obscure functional differences between processing levels. Compared with multidimensional or overly fine-grained models (e.g., Wilawan 2022), HiLoLev therefore retains two correlated factors as a parsimonious and cognitively interpretable solution that preserves theoretical distinctiveness while acknowledging shared variance.

Despite its promising results, the HiLoLev model should be interpreted with caution. The model was derived and tested in a single national context with upper-secondary students, and the final 11-item structure was developed post hoc. Because the SORS was administered in Swedish translation, translation or cultural influences on item interpretation cannot be ruled out. The present study adopted a confirmatory CFA approach consistent with its theory-driven aims; other psychometric frameworks (e.g., bifactor CFA, ESEM, or IRT) may be explored in future research. While guided by theory, the HiLoLev model has not yet been cross-validated. Future work should examine generalisability across groups, educational levels, and linguistic contexts and, where translations are used, consider measurement invariance to ensure comparable interpretation across versions, without presupposing non-equivalence. Moreover, some items excluded from the final model may prove useful in other settings. A logical next step is to test whether HiLoLev yields stronger and more consistent links to outcomes (reading comprehension, vocabulary, academic achievement) than the original subscales.

From a pedagogical perspective, the HiLoLev distinction may be used as a reflective framework rather than as a measurement tool. Learners who report extensive reliance on lower-level strategies (e.g., dictionary use, paraphrasing) may be encouraged to reflect on which local processing difficulties they are attempting to resolve, such as lexical access or syntactic parsing. Conversely, learners for whom lower-level processing is more automatised may benefit from focusing on strategies that support higher-level comprehension, such as building global coherence, integrating information across texts, and drawing inferences. In this way, the higher–lower distinction can support metacognitive awareness and differentiated instructional focus without presupposing formal assessment. At the same time, the full set of SORS items remains pedagogically valuable, as they describe recognisable actions that can be practised and reflected upon in ESL classrooms, for example in relation to planning, vocabulary support, or attention regulation, and can be used by teachers as thematic prompts for strategy-focused instruction.

From a methodological standpoint, however, using the SORS as a research instrument requires stricter validation. As shown in this study, assuming that all 30 items represent coherent and distinct constructs is unwarranted without empirical confirmation. Particularly with translated or adapted versions, researchers should verify structure (e.g., CFA; and, where relevant, basic invariance checks) before computing subscale means. Using unvalidated scales in regression analyses or structural equation modelling introduces substantial risk of bias and misinterpretation.

In conclusion, while the SORS has long served as a practical and pedagogically intuitive tool for exploring ESL students’ strategic reading behaviors, this study shows that the SORS triplet (GLOB/PROB/SUP) does not hold under confirmatory testing in this sample, and therefore should not be treated as latent subscales for regressions, group comparisons, or mediation. A compact, theory-based two-factor solution (HiLoLev) shows good fit and clearer interpretability, offering a more robust latent structure for cognitively grounded analyses of L2 readers’ strategy use. The pedagogical strengths of the SORS items remain; for inferential use, either confirm the structure in-sample via CFA or, as indicated here, consider using the 11-item HiLoLev model.

## Data Availability

The datasets generated and analysed during the current study are not publicly available due to privacy and data-protection constraints but are available from the corresponding author on reasonable request.

## SORS items: original categorisation and wording

**Table:**

Item	Category	SORS item wording
q1	GLOB	I have a purpose in mind when I read
q2	SUP	I take notes while reading to help me understand what I read
q3	GLOB	I think about what I know to help me understand what I read
q4	GLOB	I take an overall view of the text to see what it is about before reading it
q5	SUP	When text becomes difficult, I read aloud to help me understand what I read
q6	GLOB	I think about whether the content of the text fits my reading purpose
q7	PROB	I read slowly and carefully to make sure I understand what I am reading
q8	GLOB	I review the text first by noting its characteristics like length and organization
q9	PROB	I try to get back on track when I lose concentration
q10	SUP	I underline or circle information in the text to help me remember it
q11	PROB	I adjust my reading speed according to what I am reading
q12	GLOB	When reading, I decide what to read closely and what to ignore
q13	SUP	I use reference materials (e.g., a dictionary) to help me understand what I read
q14	PROB	When text becomes difficult, I pay closer attention to what I am reading
q15	GLOB	I use tables, figures, and pictures in text to increase my understanding
q16	PROB	I stop from time to time and think about what I am reading
q17	GLOB	I use context clues to help me better understand what I read
q18	SUP	I paraphrase (restate ideas in my own words) to better understand what I read
q19	PROB	I try to picture or visualize information to help remember what I read
q20	GLOB	I use typographical features like boldface and italics to identify key information
q21	GLOB	I critically analyze and evaluate the information presented in the text
q22	SUP	I go back and forth in the text to find relationships among ideas in it
q23	GLOB	I check my understanding when I come across new information
q24	GLOB	I try to guess what the content of the text is about when I read
q25	PROB	When text becomes difficult, I reread it to increase my understanding
q26	SUP	I ask myself questions I like to have answered in the text
q27	GLOB	I check to see if my guesses about the text are right or wrong
q28	PROB	I try to guess the meaning of unknown words or phrases
q29	SUP	When reading, I translate from English into my native language
q30	SUP	When reading, I think about information in both English and my mother tongue

*Tag key* q = item question, *GLOB* global; *PROB* problem-focused; *SUP* support

## Descriptive statistics for the 30 SORS items

**Table:**

Item	*N*	M	SD	Skewness	Excess kurtosis
q1	552	2.828	1.186	0.237	-0.771
q2	548	2.130	1.030	0.826	0.305
q3	554	3.621	1.166	-0.817	-0.137
q4	552	2.928	1.337	0.028	-1.228
q5	548	2.473	1.283	0.466	-0.920
q6	541	2.861	1.218	0.143	-0.991
q7	550	3.422	1.063	-0.255	-0.703
q8	547	2.366	1.294	0.622	-0.759
q9	548	3.746	0.984	-0.547	-0.076
q10	547	2.442	1.171	0.519	-0.586
q11	547	3.728	1.081	-0.635	-0.264
q12	546	2.714	1.187	0.256	-0.851
q13	552	2.592	1.270	0.399	-0.933
q14	551	3.702	1.132	-0.669	-0.324
q15	552	2.647	1.327	0.345	-1.082
q16	550	3.362	1.043	-0.156	-0.730
q17	542	3.690	1.131	-0.638	-0.367
q18	554	2.159	1.081	0.869	0.134
q19	540	2.772	1.248	0.133	-0.994
q20	544	3.480	1.151	-0.501	-0.511
q21	543	2.808	1.161	0.108	-0.887
q22	533	3.439	1.137	-0.380	-0.653
q23	534	3.476	1.037	-0.369	-0.455
q24	539	2.894	1.243	0.154	-0.970
q25	538	3.154	1.236	-0.182	-0.964
q26	535	2.490	1.132	0.433	-0.604
q27	530	2.981	1.260	0.001	-1.035
q28	545	3.461	1.113	-0.225	-0.921
q29	486	2.397	1.286	0.587	-0.826
q30	488	3.258	1.297	-0.261	-1.000

*N*s vary due to item-level missingness. *M* and *SD* are based on the 1–5 Likert scale. Skewness and kurtosis are computed from raw item scores; kurtosis is reported as excess kurtosis. For CFA with MLR, rule-of-thumb thresholds are |skewness| ≤ 2 and |excess kurtosis| ≤ 7. In this sample, skewness ranged from − 0.82 to 0.87 and excess kurtosis from − 1.23 to 0.31—well within those ranges

## Two-factor CFA results for all 30 items (HILES vs. LOLES) and pruning rationale

**Table:**

Item	A-priori factor	Loading STDYX	Item *R*²	Diagnostics & decision rationale
q1	HILES	0.403	0.162	WL/WR²; LD with q3 (resid z ≈ 7.4); PAR—dropped in favor of stronger HILES anchors
q2	LOLES	0.644	0.415	ANCHOR; strong, clean indicator; no substantive CL/LD after pruning
q3	HILES	0.542	0.293	ANCHOR; generally clean; LD with q1 handled by dropping q1; minor ties to q20 attenuated after pruning
q4	HILES	0.532	0.283	Cross-factor signals to LOLES items (e.g., q5 z ≈ 3.3; q10 z ≈ 3.2; q13 z ≈ 3.7); LD with q8 (z ≈ 5.8) & q20 (z ≈ 7.6); PAR—kept q20 instead
q5	LOLES	0.502	0.252	Retained; LD with q16 (z ≈ 5.6) dissipates when q16 is removed
q6	HILES	0.456	0.208	Modest λ; LD with q7 (z ≈ − 3.9); PAR—coverage subsumed by stronger HILES anchors ⇒ excluded
q7	LOLES	0.482	0.233	LD with q16 (z ≈ 6.5) and q6 (z ≈ − 3.9); PAR—LOLES captured by q2/q5/q10/q13/q18
q8	HILES	0.423	0.179	WL/WR²; LD with q4 (z ≈ 5.8); PAR—overlap with q4/q20
q9	HILES	0.457	0.209	ANCHOR; wording overlap shows as LD with q14 (z ≈ 7.6) but content is distinct; acceptable
q10	LOLES	0.588	0.346	ANCHOR; modest cross-loading signal onto HILES in MLR (HILES BY q10, MI ≈ 13.7) but acceptable; LD with q22 (z ≈ 5.6) resolved by dropping q22
q11	HILES	0.424	0.180	Retained for conceptual coverage; mostly clean (e.g., moderate LD with q23 z ≈ 4.6)
q12	HILES	0.411	0.169	WL/WR²; minor LDs (e.g., with q24 z ≈ 3.8; with q22 z ≈ 2.2); PAR
q13	LOLES	0.595	0.354	ANCHOR; LD with q30 (z ≈ − 3.9) resolved by dropping q30
q14	HILES	0.522	0.272	ANCHOR; MLR MIs suggest small cross-loading to LOLES in the short model (LOLES BY q14, StdYX EPC ≈ − 0.16); LD with q9 (z ≈ 7.6) acceptable given distinct emphasis
q15	HILES	0.369	0.136	WL/WR²; LD with q20 (z ≈ 4.2) and q30 (z ≈ 3.3); PAR ⇒ excluded
q16	HILES	0.485	0.235	Ambiguous cross-factor ties (e.g., q5 z ≈ 5.6; q13 z ≈ 4.3; q2 z ≈ 2.7) + LD with q7 (z ≈ 6.5) ⇒ excluded
q17	HILES	0.513	0.264	Cross-factor indications (e.g., q18 z ≈ 3.6); LD with q26 (z ≈ − 4.4) and q16 (z ≈ 5.7); PAR—overlaps with q20/q21
q18	LOLES	0.456	0.208	Retained; LD with q29 (z ≈ 5.2) resolved by dropping q29; small CL signal in short model (HILES BY q18, MI ≈ 4.4) is minor
q19	LOLES	0.455	0.207	Adequate λ but PAR—adds little beyond S5/S10; lower R² than S10 (0.207 vs. 0.346)
q20	HILES	0.566	0.320	ANCHOR; strong; LD with q4 (z ≈ 7.6) and modest ties to q26 (z ≈ − 2.6) handled by pruning those items
q21	HILES	0.530	0.281	ANCHOR; LD with q26 (z ≈ 6.2) removed by dropping q26
q22	HILES	0.579	0.335	Cross-factor entanglement/overlap with LOLES (LD with q10 z ≈ 5.6); PAR—S10 retained as LOLES anchor
q23	HILES	0.620	0.384	Strong λ but LD with q27 (z ≈ 6.3) + several smaller LDs; PAR—coverage retained via q20/q21
q24	HILES	0.400	0.160	WL/WR²; LD with q28 (z ≈ 6.4); PAR
q25	HILES	0.475	0.225	Redundant with stronger HILES items; LD signals (e.g., with q4 z ≈ − 4.1; q14 z ≈ 4.3); PAR
q26	HILES	0.487	0.237	Extensive LD across both factors (e.g., with q21 z ≈ + 6.2, q17 z ≈ − 4.4, q7 z ≈ − 3.1, q10 z ≈ + 3.0, q20 z ≈ − 2.6); promotes local misfit ⇒ excluded
q27	HILES	0.525	0.275	LD with q23 (z ≈ 6.3); minor cross-factor ties; PAR
q28	HILES	0.263	0.069	WL/WR²; LD with q24 (z ≈ 6.4); PAR
q29	LOLES	0.339	0.115	WL/WR²; LD with q30 (z ≈ 6.1) & q18 (z ≈ 5.2) and cross-factor ties (e.g., q23 z ≈ − 3.5); excluded
q30	LOLES	0.262	0.068	WL/WR²; LD with q29 (z ≈ 6.1) & q13 (z ≈ 3.9); excluded

*Tag key*: ANCHOR = retained, clean, conceptually central indicator. WL = weak loading (λ < 0.45). WR² = weak item R² (< 0.20). CL = cross-loading tendency (direction). LD = local dependence (pair; standardised residual z≈). PAR = parsimony/overlap

Pruning refers to the stepwise removal of redundant or unstable items to achieve a parsimonious and interpretable final model

## Footnotes

1 The terms used in the work by Mokhtari, Sheorey and colleagues are most often referred to as ’native’ vs. ’non-native’ speakers of English. However, throughout this paper the terms ’first language’ (L1) vs. ’second language’ (L2) are used instead.
