# Correction: Higher- and lower-level processing in strategic reading: Reconceptualising the Survey of Reading Strategies (SORS)

**DOI:** 10.1007/s10339-026-01385-x

**Published:** 2026-08-08

**Authors:** Marcus Warnby

**Affiliations:** Department of Education and Special Education, University of Gothenburg, Gothenburg, Sweden

**Correction: Cognitive Processing (2026)** https://doi.org/10.1007/s10339-026-01372-2

In this article, the last line of the paragraph was incorrect in the Method – Instruments section which previously appeared as

“Item wording is given in Appendix 1, and descriptive statistics for the three SORS subscales derived from the full 30-item instrument are reported in Appendix 2.”

But should have been

“Item wording is given in Appendix 1, and descriptive statistics for all 30 SORS items are reported in Appendix 2.”

The original article has been corrected.

